# Global Associations of Air Pollution and Conjunctivitis Diseases: A Systematic Review and Meta-Analysis

**DOI:** 10.3390/ijerph16193652

**Published:** 2019-09-28

**Authors:** Renchao Chen, Jun Yang, Chunlin Zhang, Bixia Li, Stéphanie Bergmann, Fangfang Zeng, Hao Wang, Boguang Wang

**Affiliations:** 1 Institute for Environmental and Climate Research, Jinan University, Guangzhou 511443, China; chenrenchao_jnu@163.com (R.C.); zhchunlin@163.com (C.Z.); libixia_jnu@163.com (B.L.); haowang201314@126.com (H.W.); 2 Faculty of Health, Medicine and Life Sciences, Maastricht University, Maastricht 6200 MD, The Netherlands; sat.bergmann@student.maastrichtuniversity.nl; 3 Department of Epidemiology, School of Medicine, Jinan University, Guangzhou 510630, China; zengffjnu@126.com

**Keywords:** air pollution, conjunctivitis disease, vulnerable populations, systematic review and meta-analysis

## Abstract

(1) Background: As the most common eye disease diagnosed in emergency departments, conjunctivitis has caused serious health and economic burdens worldwide. However, whether air pollution may be a risk factor for conjunctivitis is still inconsistent among current evidence. (2) Methods: We searched the literature on the relationship between air pollution and conjunctivitis in multiple English databases before 18 March 2019. Meta-analysis, meta-regression, and funnel plots were used to integrate the data, identify the sources of bias, and determine the publication bias, respectively. (3) Results: A total of 2450 papers were found, 12 of which were finally included. The pooled relative risk for each 10 μg/m^3^ increase of air pollution on conjunctivitis was 1.0006 (95%CI: 0.9993–1.0019) for CO, 1.0287 (1.0120–1.0457) for NO_2_, 1.0089 (1.0030–1.0149) for O_3_, 1.0004 (0.9976–1.0032) for PM_2.5_, 1.0033 (0.9982–1.0083) for PM_10_, and 1.0045 (0.9908–1.0185) for SO_2_. In the subgroup, PM_2.5_ and O_3_ had a greater impact on conjunctivitis risk in women than in men, and people <18 years old than those ≥18 years old. Relative humidity significantly modified the risk of O_3_ on conjunctivitis (*p* = 0.023), explaining 45% of the between-study heterogeneity. (4) Conclusion: Globally, air pollution has considerable health risks for conjunctivitis. Females and the youth were more vulnerable to PM_2.5_, NO_2_, and O_3_. Reductions of air pollution levels are still warranted to protect the vulnerable populations.

## 1. Introduction

Ambient air pollution is one of the most important risk factors that affects people worldwide [1,2,3]. Numerous epidemiological investigations have revealed the short-term or long-term associations between high concentrations of air pollutants and increased health outcomes, including stroke, heart disease, lung cancer, diabetes, and chronic lung disease. Dense innervations in the ocular surface are extremely sensitive to environmental chemical substances. In addition, human eyes are only protected by a thin layer of tear film, causing them to be very susceptible to the harmful effect of air pollution [4,5,6]. 

Conjunctivitis disease is generally divided into two categories: infectious by pathogenic microbial factors and non-infectious by physicochemical factors. Conjunctivitis is the most common eye disease diagnosed in emergency departments and affects all ages, which has caused serious health and economic burdens around the world. For instance, in the United States, conjunctivitis accounts for almost one third of all eye-related diseases, with 4–6 million conjunctivitis visits annually and a treatment cost of nearly 800 million dollars [7]. In addition to the societal costs, conjunctivitis can directly influence the patients’ quality of life. Mild conjunctivitis can affect people’s learning and working, and severe conjunctivitis can cause irreversible damage to the eyes, such as decreased vision or even blindness [4]. Additionally, patients with conjunctivitis, particularly allergic conjunctivitis, always have coexisting symptoms, such as allergic asthma and rhinitis [8]. Therefore, identifying the environmental risk factors for conjunctivitis and then guiding the development of effective measures for reducing the incidence of conjunctivitis are important for public health in the field of ophthalmology. Recently, several studies have provided evidence that exposure to air pollution could significantly increase the risk of conjunctivitis development [9,10,11,12]. However, there is still significant controversy on which air pollutants pose the highest risk and which subpopulation of patients with conjunctivitis is particularly sensitive to air pollution. For example, Bourcier and colleagues [13] reported that NO_2_ is associated with a higher risk of conjunctivitis in the population than that of O_3_, while Larrieu et al. showed the opposite results [14]. In addition, Hong and co-authors found that the effect of O_3_ is greater for women with conjunctivitis than men, whereas the study of Fu et al. presented the opposite trend [5,6]. These inconsistent results indicate the necessity of quantitatively synthesizing and interpreting the current available evidence in order to provide comprehensive evidence for policymakers and protect the public’s health.

In this study, we aimed to perform a systematic review and meta-analysis to combine the global associations between air pollutants and conjunctivitis, and to identify the sensitive subgroups.

## 2. Materials and Methods 

### 2.1. Data Source

We searched for articles published before 18 March 2019 in the following electronic databases: Web of Science, PubMed, Embase, and Scopus. The search terms included “conjunctivitis,” “pinkeye,” “air pollution,” “CO,” “NO_2_,” “SO_2_,” “O_3_,” “PM_2.5_,” and “PM_10_” (see Table A1). The reference lists of the included studies were further examined for additional studies.

### 2.2. Study Selection

#### 2.2.1. Selection Criteria

The following inclusion criteria were utilized in this study:

1. Risk assessments on the relationship between air pollutants and health outcomes of conjunctivitis;

2. Studies providing quantitative effect estimates, such as the excess rate (ER), risk ratio (RR), odds ratio (OR), regression coefficient (β) or percentage and standard error (SE), and the respective 95% confidence interval (CI);

3. Literature using the following methodology: time-series, case-crossover, logistic regression, a generalized linear model (GLM), a generalized additive model (GAM), and a distributed lag model (DLM);

4. Studies that reported the link between exposures in the form of lag (day) and health outcome.

The exclusion criteria were as follows:

1. Not original research studies (e.g., commentary, communication, review, and meeting abstract);

2. Not related to outdoor air pollution (e.g., indoor, workplace, office);

3. Research related to clinical or animal experiments (e.g., drug trials, mice, and rabbits);

4. Research on conjunctivitis not caused by air pollution factors (e.g., mites and pollen);

5. The target was not conjunctivitis diseases (e.g., rhinoconjunctivitis or conjunctivitis of other organs).

#### 2.2.2. Data Extraction

Data from all included studies that were extracted were as follows: the reference, study design, demographic data (e.g., GDP and population), average values of air pollutants, meteorological variables (e.g., temperature, relative humidity, and air pressure), and effect estimates (e.g., RR, regression coefficient, 95% confidence interval, and standard error). For articles with missing information, we contacted the corresponding authors by email to obtain the relevant data.

#### 2.2.3. Quality Assessment

In order to distinguish between low-quality and high-quality studies, a quality assessment was performed. Due to the wide variety of study designs used in the literature, assessing the quality and their risk of bias can be difficult. 

To the best of our knowledge, no validated scale has been developed to assess the quality of time-series and case-crossover studies. We selected and combined several items from the New Castle Ottawa Scale [15], the Cochrane risk of bias tool, and other tools [16], which were utilized in previous studies [17,18,19]. We created a five-point scoring system that included the following four aspects:

a. Conjunctivitis disease occurrence verification (0–1 points)

According to the International Classification of Diseases, studies on the causes of death encoded in revised version 9 (ICD-9), 10th revision, or ICPC-2 Code(s) (International Classification of Primary Care, Second Edition [20]) and official definitions of other countries are given a score of 1, but no score is given for studies that do not meet the criteria.

b. Quality of air pollutant measurements (0–1 points)

The quality of the air pollutant measurement can be judged according to the measurement frequency and the existence of missing data. If the measurement is made at least once a day and the missing data is <25%, the research score is 1; otherwise, the quality is assessed with 0 points.

c. Adjustment degree of confounders (0–3 points)

Adjustment for temperature and humidity is given 1 point. Additional adjustments, for example, seasonality, wind speed, or rainfall, acquire 2 points. If the long-term trend and days of the week are considered, 3 points are given. Zero points are given if there is no adjustment for temperature and humidity. 

If the study gets full marks for all three components, the study was considered to be of a good quality. If any of the three components were zero, the study quality was considered to be low. All other studies were considered to be of a medium quality.

### 2.3. Data Synthesis and Statistical Analysis

The key objective of data synthesis was to unify the air pollutant concentration units, group the research population, and standardize the risk effect values. If studies used mg/m^3^, ppm, or ppb for the unit of measurement or unit of increment, all estimates were converted into μg/m^3^. Regarding population groupings, the included data were mainly divided into two groups: gender (male and female) and age group (>18 years old and <18 years old). In most studies, the risk estimates were expressed as ERs, ORs, or RRs with 95% CIs, and percent changes. The results presented as a regression coefficient and standard error were converted to RR. The summarized statistics are expressed as RRs with 95% CIs [21,22]. To pool the effect estimates, all estimates were standardized to an increment of 10 μg/m^3^ of air pollutant (CO, O_3_, SO_2_, NO_2_, PM_2.5_, and PM_10_) concentration.

The statistical analysis consisted of three steps: (1) computing the integrated estimates of each type of air pollutant using a fixed- or random-effect meta-analysis; (2) conducting a meta regression analysis based on the total population, GDP, and weather conditions; and (3) performing a sensitivity analysis. A meta-analysis was used to aggregate the risk estimates from all studies in detail. If the heterogeneity index (*I^2^*) was greater than 25%, the aggregate estimates were calculated using a random effect model; otherwise, we selected the fixed effect model [23]. The second step was to judge and test the source of heterogeneity. Heterogeneity was classified as high (*I^2^* > 75%), medium (25 < *I^2^* < 75%), or low (*I^2^* < 25%) [24]. The sources of heterogeneity, such as the research design, regional GDP, geographic location (longitude and latitude, temperature, and humidity), and weather conditions, were further tested using a meta-regression analysis. Finally, we applied funnel charts and Begg’s [25] and Egger’s tests [26] to assess the potential impact of publishing bias. We conducted the sensitivity analysis by re-calculating the pooled effects by excluding each study to test whether our main findings were influenced by one study. 

Statistical analysis and drawing were mainly conducted using R language software (R version 3.6.0; R Development Core Team, New Zealand, Australia).

## 3. Results

### 3.1. Search Results and Study Characteristics

**Figure 1:**
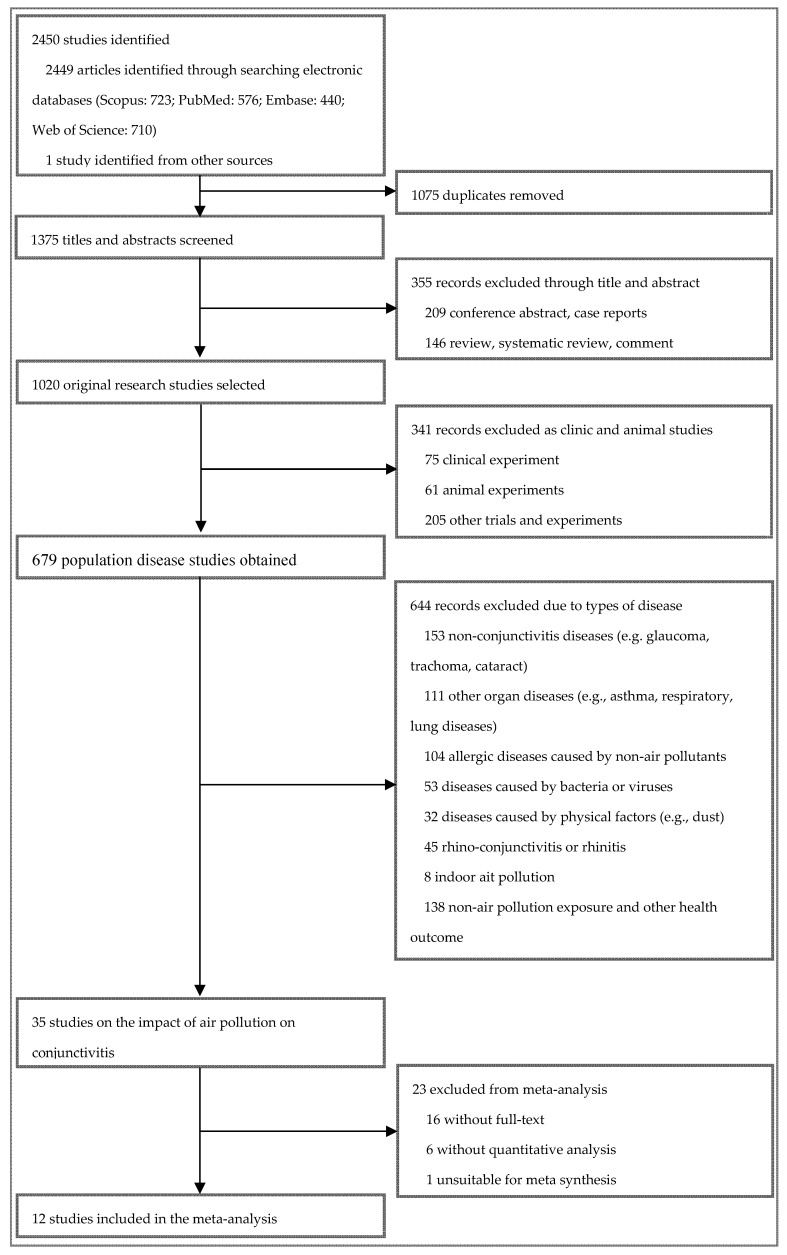
Flow chart for the study selection process.

In this study, 2450 records were originally obtained from Scopus (n = 723), PubMed (n = 576), Embase (n = 440), and Web of Science (n = 710). Twelve articles from 10 regions met the inclusion criteria and were included in the meta-analysis (see Figure 1), covering 30,103,982 conjunctivitis patients. Among the 12 included studies, five were case-crossover studies [4,5,9,27,28], four were time-series studies [6,14,29,30], and three were other studies (e.g., spatial analysis and multi-level regression). Table 1 and Table A2 summarize the basic characteristics of the included studies. The number of research papers including CO, NO_2_, O_3_, PM_2.5_, PM_10_, and SO_2_ was two, seven, nine, four, seven, and seven, respectively.

### 3.2. Overall Analysis

**Figure 2:**
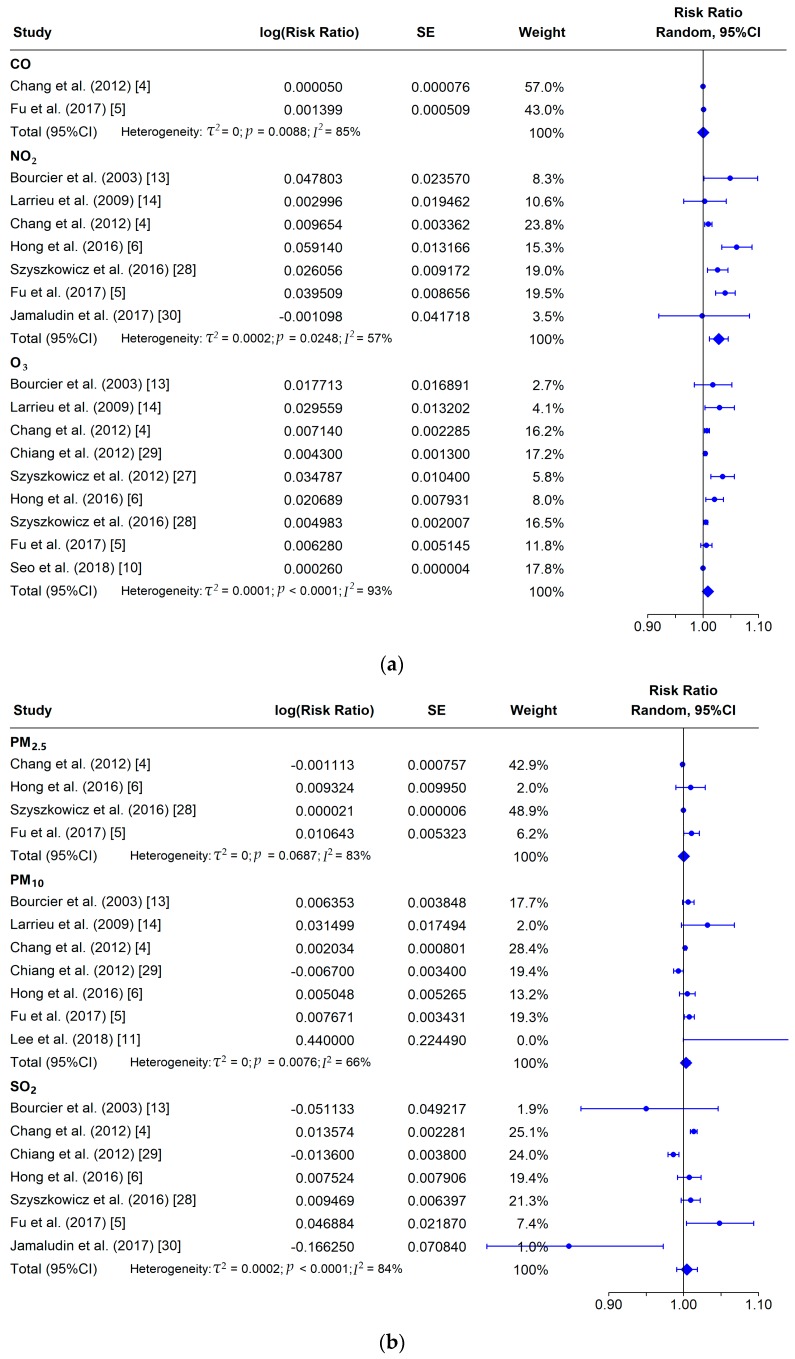
Forest plot of the association between conjunctivitis and exposure to air pollution: (**a**) CO, NO_2_, O_3_; and (**b**) PM_2.5_, PM_10_, SO_2_. Risk ratio was calculated by considering a 10 μg/m^3^ increase of air pollution.

Since significant heterogeneity (*I^2^* > 60%) was observed in the included studies, we used a random-effect meta-analysis to integrate the effect estimates of various air pollutants on conjunctivitis [31]. Figure 2 presents the pooled effect of six air pollutants on the risk of conjunctivitis among the included studies. The pooled relative risk for each 10 μg/m^3^ increase of air pollutants on conjunctivitis was 1.0006 (95%CI: 0.9993–1.0019) for CO, 1.0287 (95%CI: 1.0120–1.0457) for NO_2_, 1.0089 (95%CI: 1.0030–1.0149) for O_3_, 1.0004 (95%CI: 0.9976–1.0032) for PM_2.5_, 1.0033 (95%CI: 0.9982–1.0083) for PM_10_, and 1.0045 (95%CI: 0.9908–1.0185) for SO_2_.

### 3.3. Subgroup Analysis

Given the limited number of articles, we could only combine the effect estimates by subgroup for PM_2.5_, NO_2_, and O_3_ (Table 2). The random-effect meta-analysis was used to pool the effect risk of air pollution on conjunctivitis among subgroups as the heterogeneity was significant. Generally, the impact of air pollution was higher among females and the youth than the other groups. However, only statistically significant effects of O_3_ on males, with an RR value of 1.0321 (95%CI: 1.0000–1.0653), and NO_2_ and O_3_ on the youth, with corresponding RR values of 1.0472 (95%CI: 1.0249–1.0700) and 1.0357 (95%CI: 1.0156–1.0561), were found.

### 3.4. Meta-Regression

In order to assess the source of the between-study heterogeneity, a meta-regression was further conducted to test the influence of city-level characteristics (e.g., GDP, longitude and latitude, average temperature, relative humidity, and duration of sunshine) on the relationship between air pollution and conjunctivitis (see Table 3). Among these factors, only the relative humidity significantly modified the risk of O_3_ for conjunctivitis (*p* = 0.023), explaining 45% of the between-study heterogeneity. 

### 3.5. Publication Bias

**Figure 3:**
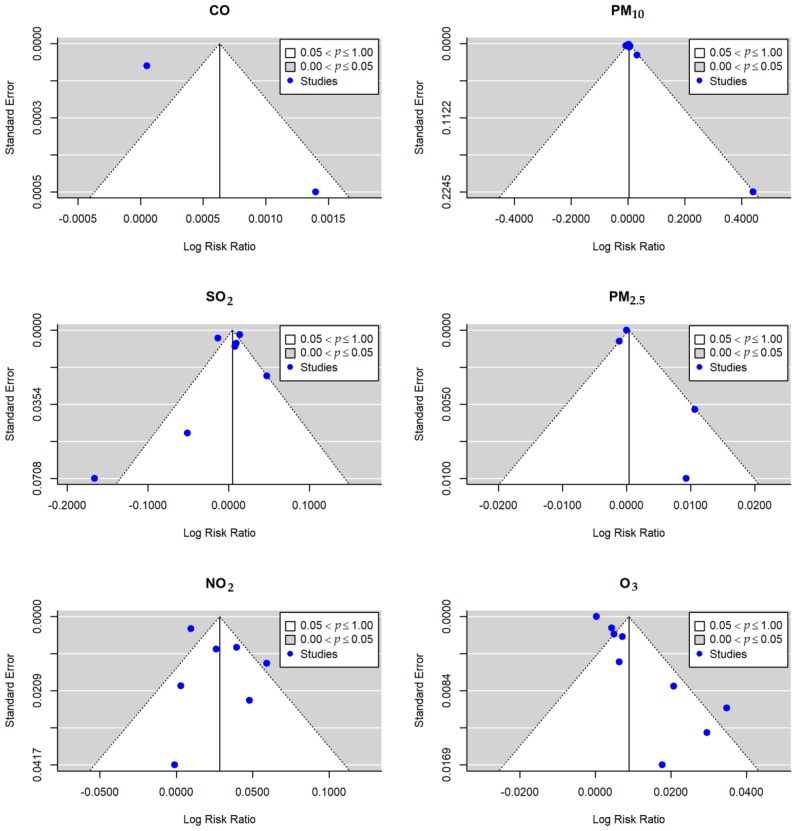
Funnel plot showing the risk of publication bias in the meta-analysis on the risk of conjunctivitis with per 10 μg/m^3^ increase of air pollutants. Horizontal axis represents the log RR and vertical axis represents standard errors.

**Figure A1:**
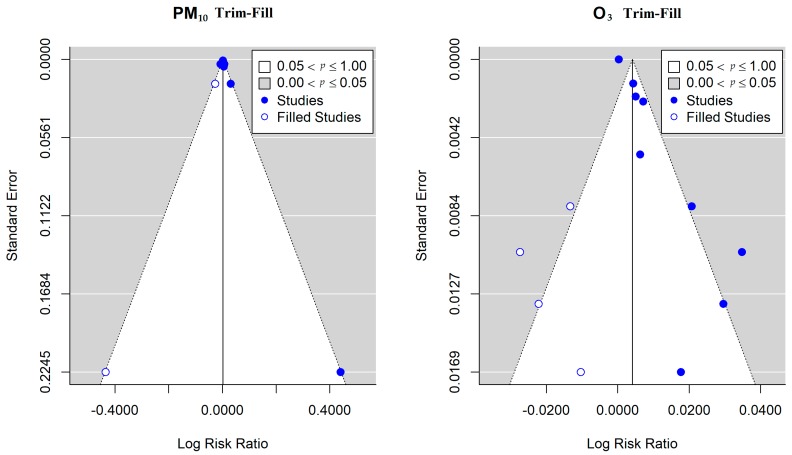
Funnel plot of PM_10_ and O_3_ on conjunctivitis using Trim-fill method. The solid circles denote effect estimates from included studies and open circles denotes estimates provided by Trim-fill method.

Funnel plot, Begg’s, and Egger’s tests were applied to determine whether there was publication bias. Figure 3 shows the funnel plots of the meta-analysis for the association between air pollution and the risk of conjunctivitis. The results of PM_2.5_, SO_2_, and NO_2_ presented a low probability of publication bias, reporting a *p*-value for both Begg’s test and Egger’s test of over 0.05. However, potential publication bias was detected for PM_10_ (Egger’s test: Z-value = 2.4238, *p* = 0.0154) and O_3_ (Egger’s test: Z-value = 5.4884, *p* < 0.001) (see Table 4). In addition, we performed the trim and fill method to validate the publication bias of PM_10_ and O_3_ (see Figure A1). The adjusted pooled relative risk of PM_10_ for total conjunctivitis was 1.0026 (95%CI: 0.9975, 1.0077) and 1.0041 (95%CI: 0.9957, 1.0126) for O_3_. 

### 3.6. Sensitivity Analysis

Sensitivity analyses were performed to estimate the stability of the results by recalculating the pooled effect estimates after omitting one study each time [32,33,34]. We found that the effect estimate of each 10 μg/m^3^ increase in the six air pollutants showed no significant change by removing one single study, suggesting that the combined results were relatively stable and reliable. 

## 4. Discussion

To the best of our knowledge, this is the first systematic review and meta-analysis to assess the association between air pollution and conjunctivitis. Twelve studies, including 30,103,982 cases of conjunctivitis from 10 countries/regions around the world, were included. Positive associations between six common air pollutants and conjunctivitis were obtained, while statistical significance was only observed for NO_2_ and O_3_. The female subgroup and those under 18 years old were most vulnerable to the risk of conjunctivitis caused by air pollution.

### 4.1. Risk Analysis of Air Pollution and Conjunctivitis in the Whole Population

In the past decade, the effect of air pollution on conjunctivitis has attracted increasing interest [4,35,36]. However, the evidence so far is inconsistent (Figure 2). For instance, Fu et al. [5] revealed that the risk of NO_2_ and conjunctivitis in the population was significant, with an RR value of 1.0403 (95%CI: 1.0228, 1.0581), while Jamaludin et al. [30] did not find any significant effects on the risk of conjunctivitis in the population, with an RR value of 0.9989 (95%CI: 0.9205, 1.0840). For PM_10_, Chang et al.’s [4] study revealed that PM_10_ was significantly associated with the conjunctivitis risk among people, with an RR value of 1.0020 (95%CI: 1.0005, 1.0036). However, in the study of Chiang et al. [29], NO_2_ had no significant effect on the risk of conjunctivitis in people, with an RR value of 0.9933 (95%CI: 0.9867, 1.0000). For SO_2_, Fu et al.’s study [5] revealed that the risk of conjunctivitis between SO_2_ and the population was significant, with an RR value of 1.0480 (95%CI: 1.0040, 1.0939). In the study of Jamaludin et al. [30], SO_2_ had a protective effect on the conjunctivitis risk among people, with an RR value of 0.8468 (95%CI: 0.7371, 0.9730). Air pollution is gradually occupying an important position in the risk factors of conjunctivitis. Our study shows that all six air pollutants have a positive correlation with conjunctivitis. Among them, NO_2_ had the most significant effect, followed by O_3_. This may be due to differences in the physical and chemical properties between pollutants, resulting in different risk outcomes. Both NO_2_ and O_3_ are highly oxidative and irritating to the eyes [37,38,39,40]. According to the chemical properties of O_3_ and NO_2_, O_3_ is easily removed by a reaction, so the lifetime of NO_2_ is longer than that of O_3_ [41,42]. In addition, in terms of toxicity, the toxicity of O_3_ may be more complex than that of NO_2_ [43,44], which may have a significant potential impact on eye tissue cells. From the comprehensive analysis of the toxicity degree and lifetime of pollutants, NO_2_ and O_3_ have obvious risks for conjunctivitis in the population, among which, NO_2_ has the highest risk value, followed by O_3_.

### 4.2. Risk Analysis of Air Pollution and Conjunctivitis in Subgroups

According to the research analysis, PM_2.5_, NO_2_, and O_3_ present a higher risk for conjunctivitis in women than in men; meanwhile, PM_2.5_ and O_3_ exhibit a higher risk for conjunctivitis for people under 18 years of age than people over 18 years of age, whereas NO_2_ had the opposite effect. Between genders, there are three possible reasons for the greater risk of conjunctivitis in women. First, women’s physical function is generally not as good as men’s [45], so their ability to resist air pollution is relatively weak. Second, women spend more time indoors than men [46,47], and indoor air circulation is not strong, so more toxic and harmful air pollutants may more easily accumulate and then be absorbed. Third, compared with men, women prefer makeup [48], especially eye shadows, eyelashes, and contact lenses. Studies have shown that these types of eye makeup can cause discomfort to the eyes, such as dryness, pain, etc. [49,50,51,52], which may increase the risk of conjunctivitis. Therefore, in combination with the above points, the risk for females of conjunctivitis is greater than that for males. In terms of the age group, for people younger than 18 years old, the development of physical function and the defense ability is still immature and they are thus vulnerable to air pollutants. The effects of NO_2_ on people over 18 years of age was significantly greater than that on people under 18 years of age, which may be related to people’s living and working habits. People over the age of 18 go to work, which often involves the need to travel between cities, so there is a relatively high chance of exposure to severe air pollution scenarios [53]. Exposure to more mobile sources of pollution, such as NO_2_ emitted by automobiles [54], increases the risk of conjunctivitis in adults.

### 4.3. Source of Heterogeneity and Possible Bias

For GDP, latitude, longitude, temperature, and humidity, we observed substantial heterogeneity in the pooled effect sizes of air pollutants (NO_2_, O_3_, PM_2.5_, PM_10_, and SO_2_) for conjunctivitis. We found that there was a negative correlation between relative humidity and the risk of conjunctivitis for five kinds of air pollutants. There may be several explanations for this.

First, the higher the humidity in the air, the easier it is to condense and settle the solid particles in the air [55], and the easier it is to dilute the liquid or gaseous pollutants. These processes can reduce the concentration of pollutants in the air, thereby reducing the risk of conjunctivitis. Second, a high humidity will affect visibility [56], which will affect people’s travel habits; therefore, to a certain extent, it can reduce the risk of exposure to conjunctivitis. Finally, from a physiological point of view, in greater humidity, the eyes will be relatively comfortable (so it is not easy to itch the eyes, not easy to rub the eyes, etc.) and thus dry eye will not be easily caused [57]. Furthermore, it reduces the risk of conjunctivitis.

### 4.4. Possible Mechanisms Explaining the Relation between Conjunctivitis and Air Pollution

To date, the underlying pathophysiological mechanism of conjunctivitis caused by air pollutants is still unclear. As a human’s eyes are directly exposed to air pollution, some studies have speculated that PM_2.5_ [35,58,59] and PM_10_ [60] particles may easily cause the inadaptability of intraocular epidermal cells, leading to cell death and the inflammation of tissue cells. Second, NO_2_ and O_3_ have strong oxidative stress effects [61], which may stimulate conjunctival cell inflammation. Finally, NO_2_ is an acidic gas. When it enters the eyes, it easily changes the acidic and alkaline environment of the inner epidermis cells of the eyes [62], breaking the function of the eye cells and causing inflammation [63,64]. It is plausible that the association between air pollution and the risk of conjunctivitis events is a result of these important mechanistic pathways.

### 4.5. Limitations and Implications

Several limitations of our study should be considered. First, almost all the included references used the air pollutant data from fixed environmental monitoring stations instead of individual-level air pollutant exposures, which may have led to measurement error. Second, we included studies in the same place at different times (for example, Taiwan), which may have also had an impact on the combined value of conjunctivitis risk. Finally, few studies were available on the association between some types of air pollutants (e.g., carbon monoxide) and the risk of conjunctivitis, which led to a relatively low statistical power and limited the further stratified assessment for subgroups. Therefore, future epidemiological evidence from more countries and/or cities with a well-designed strategy is required to be able to develop more comprehensive knowledge on the effect of air pollution on the risk of conjunctivitis. Further investigations are also needed to identify the subgroups that are most vulnerable to air pollution, and the socioeconomic status should be considered. It would also be useful to explore the use of alternative exposure metrics that are more representative of individual exposure, and it would be beneficial to examine the mechanism underlying the harmful effect of air pollution on patients with conjunctivitis. Additionally, a cost-effectiveness of preventive measures for improving the air quality to reduce the incidence of conjunctivitis is also needed in future research. 

## 5. Conclusions

This meta-analysis found that air pollution is an important factor for the risk of conjunctivitis. NO_2_ presented the highest impact on patients with conjunctivitis, followed by O_3_. For different sub-groups of patients with conjunctivitis, females and the age group under 18 years old were more sensitive to the air pollution. Notable inconsistencies in the various studies have been found for the association between air pollution and conjunctivitis, while only relative humidity significantly modified the risk of O_3_ for conjunctivitis, which explained 45% of the between-study heterogeneity. Our findings highlight the necessity for the reduction of air pollution levels and protection of vulnerable populations. Further research is needed to better understand the mechanisms underlying the harmful effect of air pollutants on the risk of conjunctivitis. Future well-designed epidemiological studies from more countries and/or cities are still warranted to be able to get more comprehensive knowledge and powerful evidence about the effect of air pollution on the risk of conjunctivitis and identification of the subpopulations sensitive to air pollution. 

## Figures and Tables

**Table 1:** The characteristics of studies included in the meta-analysis.

Study	Location	Study Design Time-span	Study Population	Pollutant	Controlled Variables	Total Events	Lag (d/w)	Main Findings
**Bourcier et al. (2003)** [13]	Paris, France	Logistic regression 31/1/1999–31/12/1999	All	NO, NO_2_, O_3_, SO_2_, PM_10_	Temperature, pressure, humidity, wind speed, day of the week	1272	d: 0–2	A strong relation between NO, NO_2_, and conjunctivitis was observed. Atmospheric pressure, minimal humidity, and wind speed may increase the incidence of ocular surface complaints.
**Larrieu et al. (2009)** [14]	Bordeaux, France	Time series Poisson regression model 2000–2006	All	NO_2_, PM_10_, O_3_	Long-term trends, seasonality, days of the week, holidays, temperature, influenza epidemics	179,142	d: 0–3	There was a much higher effect of nitrogen dioxide on visits for conjunctivitis when delayed effects were considered. Conjunctivitis was also significantly associated with PM_10_ and ozone levels.
**Chang et al. (2012)** [4]	Taiwan, China	Case-crossover Meta-analysis 2007–2009	All	CO, NO_2_, SO_2_, O_3_, PM_10_, PM_2.5_	Temperature, rainfall, humidity	26,314,960	d: 0, 0–1 to 0–5	The effects on outpatient visits for nonspecific conjunctivitis were strongest for O_3_ and NO_2_. In winter, PM_10_ and SO_2_ had a more prominent impact on the risk of conjunctivitis.
**Chiang et al. (2012)** [29]	Taiwan, China (four cities)	Time series Generalized linear model 2000–2007	All	PM_10_, SO_2_, NO_x_, O_3_	Relative humidity, wind speed, rainfall, public holiday, calendar months and years.	234,366	d: 0	There were higher risks of conjunctivitis in rural areas, but higher sensitization to air pollutants in urban cities. Children, females, and the older population were at higher risks for both types of conjunctivitis.
**Szyszkowicz et al. (2012)** [27]	Edmonton, Canada	Case-crossover Logistic regression Time-stratification 1/4/1992–31/3/2002	All, Sex: male, female	O_3_	Long-term trends, seasonal effects, day-of-week and month-of-year effects	7526	d: 3–8	For conjunctivitis, associations of these conditions with ozone exposure were observed only in females.
**Hong et al. (2016)** [6]	Shanghai, China	Time series Generalized least squares 2008–2012	All, Sex: male, female Age: <18, 19–40, 41–60, >60 years	SO_2_, NO_2_, PM_10_, PM_2.5_, O_3_	Periodic trends	3,211,820	w: 1, 3	Research revealed that higher levels of ambient NO_2_, O_3_, and temperature increased the chances of outpatient visits for allergic conjunctivitis. Meanwhile, those older than 40 years were only affected by NO_2_ levels.
**Szyszkowicz et al. (2016)** [28]	Ontario, Canada (nine cities)	Case-crossover Time-stratified Apr 2004–Dec 2011	All, Sex: male, female Age: ≤17, ≥18 years	NO_2_, O_3_, SO_2_, PM_2.5_	Temperature, humidity	77,439	d: 0–8	There were positive associations between air pollution and ED visits for conjunctivitis, with different temporal trends and strength of association by age, sex, and season. Children and young adults were more vulnerable to conjunctivitis infections.
**Fu et al. (2017)** [5]	Hangzhou, China	Time-stratified Case-crossover Logistic regression 1/7/2014–30/6/2016	All, Sex: male, female Age: 0–1, 2–5, 6–18, 19–64, >65 years	PM_10_, PM_2.5_, SO_2_, NO_2_, O_3_, CO	Temperature, humidity, atmospheric pressure	9737	d: 0, 0–1	PM_10_, PM_2.5_, SO_2_, NO_2_, and CO were associated with the risk of conjunctivitis. SO_2_ was significantly associated with conjunctivitis patients between 2 and 5 years old and male. PM_10_ and NO_2_ were significantly associated with female conjunctivitis patients.
**Jamaludin et al. (2017)** [30]	Johor Bahru, Malaysian	Time series Poisson generalized linear model, negative binomial model 1/1/2012–31/12/2013	All	NO_2_, PM_10_, SO_2_	Rainfall, temperature, humidity	1396	w: 14,19,20	SO_2_ was the most abundant source that contributed to the eye diseases.
**Lee et al. (2018)** [11]	Daegu, Korea	Spatial analysis 1/6/2006–31/12/2014	All	PM_10_	SO_2_, NO_2_, O_3_, CO	769	d: 0	Incidence of conjunctivitis and keratitis varied from region to region.
**Seo et al. (2018)** [10]	Seoul, South Korea	Multi-level regression model 1/1/2011–31/12/2013	All	O_3_	Temperature, humidity sex, age	48,344	d: 0	The outpatient incidence of conjunctivitis was increased by O_3_.
**Szyszkowicz et al. (2019)** [9]	Edmonton, Canada	Case-crossover Time-stratified Logistic regression Apr 1992–Mar 2002	Sex: male, female	O_3_	Temperature, humidity	17,211	d: 0–9	Significant association was observed for air pollution at lag 5 day for males, and lag 1 day and lag 3 day for females.

Note: d, day; w, week; CO, carbon monoxide; NO_2_, nitrogen dioxide; SO_2_, sulfur dioxide; O_3_, ozone; PM_2.5_, particles smaller than 2.5 μm; PM_10_, particles smaller than 10 μm.

**Table 2:** Risk analysis of air pollutants on patients with conjunctivitis, stratified by gender and age group.

Pollutant	Groups	No. of the Studies	Heterogeneity, *τ^2^*	Heterogeneity, *p*-value	Heterogeneity, *I^2^* (%)	Summary RR (95%CI)	*p*-Value
PM_2.5_	Male	2	0.000013	0.2131	35.5	1.0016(0.9951–1.0081)	0.6357
	Female	2	0.000028	0.1102	60.8	1.0030(0.9943–1.0117)	0.5050
	<18year	2	0.000224	0.0940	64.3	1.0086(0.9845–1.0332)	0.4877
	≥18year	2	0.000018	0.1356	55.1	1.0022(0.9952–1.0093)	0.5324
NO_2_	Male	3	0.010419	0.0001	98.4	1.0784(0.9571–1.2151)	0.2152
	Female	3	0.032345	0.0001	99.6	1.1401(0.9233–1.4077)	0.2231
	<18year	3	0.000161	0.2031	42.4	1.0472(1.0249–1.0700)	<0.0001
	≥18year	3	0.021135	0.0011	99.5	1.1128(0.9371–1.3214)	0.2228
O_3_	Male	5	0.000874	0.0083	88.2	1.0321(1.0000–1.0653)	0.0503
	Female	4	0.003334	0.0004	88.8	1.0694(0.9970–1.1471)	0.0606
	<18year	3	0.000200	0.0160	72.1	1.0357(1.0156–1.0561)	0.0005
	≥18year	3	0.000581	0.0259	93.3	1.0178(0.9879–1.0487)	0.2458

Note: RR—relative risk; CI—confidence interval.

**Table 3:** Meta-regression analysis of study level predictors on the association between air pollution and risk of conjunctivitis.

Air pollutants	Covariant	IQR	Estimate	*p*-Value	*τ^2^*	*I^2^*	*R* ^2^
NO_2_	GDP	343.07	0.24 (−2.69, 3.26)	0.873	0.000385	78.586981	0.00
	Latitude	19.21	0.57 (−2.25, 3.47)	0.695	0.000350	72.085796	0.00
	Longitude	119.96	0.44 (−2.19, 3.14)	0.745	0.000383	75.242153	0.00
	Temperature	4.28	−0.43 (−2.25, 1.42)	0.644	0.000497	85.336819	0.00
	Humidity Duration of sunshine	3.26 0.82	−2.02 (−4.35, 0.37) −2.77 (−5.60, 0.16)	0.097 0.063	0.000194 0.000188	75.394247 63.712071	44.37 33.48
O_3_	GDP	246.99	−0.65 (−1.46, 0.17)	0.120	0.000054	92.101803	0.00
	Latitude	18.61	0.70 (−0.51, 1.91)	0.259	0.000068	93.591351	0.00
	Longitude	122.10	−0.55 (−1.37, 0.28)	0.193	0.000056	93.062946	0.00
	Temperature	11.19	−0.70 (−1.83, 0.44)	0.227	0.000056	84.472565	0.00
	Humidity Duration of sunshine	4.98 0.76	−0.76 (−1.42, −0.10) 0.42 (−0.67, 1.52)	0.023 0.455	0.000009	45.134099 90.739265	0.00
					0.000073		0.00
PM_2.5_	GDP	238.83	−0.40 (−1.06, 0.27)	0.238	0.000018	66.205320	0.00
	Latitude	7.01	−0.07 (−0.58, 0.44)	0.786	0.000047	63.714683	0.00
	Longitude	52.65	0.11 (−0.29, 0.51)	0.600	0.000042	65.152911	0.00
	Temperature	4.28	0.00 (−0.55, 0.55)	0.995	0.000049	62.464757	0.00
	Humidity Duration of sunshine	3.26 0.53	−0.17 (−1.32, 0.99) −0.52 (−1.25, 0.21)	0.771 0.163	0.000030 0.000013	63.120757 69.255955	0.00 0.00
PM_10_	GDP	266.31	−0.71 (−2.00, 0.60)	0.284	0.000033	69.797637	0.00
	Latitude	12.71	0.51 (−0.21, 1.22)	0.165	0.000021	59.017623	8.35
	Longitude	60.26	-0.38 (−1.05, 0.30)	0.278	0.000027	67.965036	0.00
	Temperature	6.13	−0.73 (−1.77, 0.32)	0.171	0.000020	67.213050	23.38
	Humidity Duration of sunshine	2.44 0.63	−0.32 (−0.85, 0.21) 0.04 (−1.34, 1.44)	0.240 0.951	0.000020 0.000039	76.922096 64.984042	20.53 0.00
SO_2_	GDP	230.65	0.20 (−2.13, 2.59)	0.865	0.000314	89.692112	0.00
	Latitude	15.03	0.99 (−1.43, 3.47)	0.425	0.000319	89.037967	0.00
	Longitude	68.46	0.01 (−1.43, 1.47)	0.994	0.000358	90.307176	0.00
	Temperature	6.58	−0.47 (−2.26, 1.35)	0.608	0.000268	91.617762	0.00
	Humidity	3.04	−0.52 (−2.10, 1.08)	0.523	0.000221	90.558627	0.00
	Duration of sunshine	0.66	−0.71 (−4.23, 2.93)	0.698	0.000380	89.840483	0.00

Note: CO—carbon monoxide; NO_2_—nitrogen dioxide; SO_2_—sulfur dioxide; O_3_—ozone; PM_2.5_—particles smaller than 2.5μm; PM_10_—particles smaller than 10μm; GDP—gross domestic product; IQR—interquartile range. The descriptive information of city-level predictors is provided in Table A2.

**Table 4:** Begg’s test, Egger’s test, and trim-fill test on the effect of air pollutants on conjunctivitis.

Air Pollutants	Begg’s Test		Egger’s Test		Trim-Fill-Begg’s Test		Trim-Fill-Egger’s Test	
	*τ*	*p*-Value	*Z*-value	*p*-Value	*τ*	*p*-Value	*Z*-value	*p*-Value
CO	1.0000	1.0000	—	—				
PM_10_	0.6190	0.0690	2.4238	0.0154	0.1715	0.5271	0.0964	0.9232
SO_2_	−0.3333	0.3813	−1.6210	0.1050				
PM_2.5_	0.0000	1.0000	1.8371	0.0662				
NO_2_	0.0476	1.0000	0.0266	0.9788				
O_3_	-0.0556	0.9195	5.4884	< 0.0001	−0.1316	0.5388	−0.0208	0.9834

Note: Egger’s test was unavailable for the CO because of the limited number of studies on the association between CO and the risk of conjunctivitis. The trim-fill test was only performed for PM_10_ and O_3_, which showed significant publication bias. CO—carbon monoxide; NO_2_—nitrogen dioxide; SO_2_—sulfur dioxide; O_3_—ozone; PM_2.5_—particles smaller than 2.5 μm; PM_10_—particles smaller than 10 μm.

## Abbreviations

CO	carbon monoxide
NO_2_	nitrogen dioxide
SO_2_	sulfur dioxide
O_3_	ozone
PM_2.5_	particles smaller than 2.5 μm
PM_10_	particles smaller than 10 μm
ER	excess rate
RR	relative risk
OR	odds ratio
β	regression coefficient
SE	standard error
CI	confidence interval
GLM	generalized linear model
GAM	generalized additive model
DLM	distributed lag model
ICD-9	International Classification of Disease, Revision 9
ICD-10	International Classification of Disease, Revision 10
ICPC-2	Code(s) International Classification of Primary Care, Second Edition
GDP	gross domestic product;

## Appendix A

**Table A1:** The search strategies used in the review.

Search Field	PubMed (MeSH terms & tiab search function)	Web of Science (TS & TI search function)	Scopus (TITLE-ABS-KEY search function)	Embase (ti,ab,kw search function)
[1]	(“conjunctivitis”[MeSH Terms] OR Conjunctivitis[Title/Abstract] OR “endophthalmitis”[MeSH Terms] OR ophthalmia[Title/Abstract] OR pinkeye[Title/Abstract] OR Pink eye[Title/Abstract])	(TS=(“conjunctivitis” OR “endophthalmitis” OR “ophthalmia” OR “pinkeye” OR “conjunctivitis” OR “Pink eye”) OR TI=(“conjunctivitis” OR “endophthalmitis” OR “ophthalmia” OR “pinkeye” OR “conjunctivitis” OR “Pink eye”))	TITLE-ABS-KEY(“conjunctivitis” OR “endophthalmitis” OR “ophthalmia” OR “pinkeye” OR “conjunctivitis” OR “Pink eye”) AND TITLE-ABS-KEY(“air pollution” OR “ambient air pollution” OR “outdoor air pollution” OR “atmospheric pollution”)	“conjunctivitis”:ti,ab,kw OR “endophthalmitis”:ti,ab,kw OR “ophthalmia”:ti,ab,kw OR “pinkeye”:ti,ab,kw OR “pink eye”:ti,ab,kw
[2]	(“air pollution”[MeSH Terms] OR air pollution[Title/Abstract] OR ambient air pollution[Title/Abstract] OR outdoor air pollution[Title/Abstract] OR atmospheric pollution[Title/Abstract])	(TS=(“air pollution” OR “ambient air pollution” OR “outdoor air pollution” OR “atmospheric pollution”) OR TI=(“air pollution” OR “ambient air pollution” OR “outdoor air pollution” OR “atmospheric pollution”))	TITLE-ABS-KEY(“conjunctivitis” OR “endophthalmitis” OR “ophthalmia” OR “pinkeye” OR “conjunctivitis” OR “Pink eye”) AND TITLE-ABS-KEY( “PM_2.5_” OR “Particulate Matter2.5” OR “particulate matter” OR “PM_10_” OR “Particulate Matter 10” OR “SO_2_” OR “Sulfur dioxide” OR “NO_2_” OR “Nitrogen dioxide” OR “NO_x_” OR “Nitrogen oxides” OR “O_3_” OR “ozone” OR “CO” OR “Carbon monoxide” OR “Smog” OR “black carbon”)	“air pollution”:ti,ab,kw OR “ambient air pollution”:ti,ab,kw OR “outdoor air pollution”:ti,ab,kw OR “atmospheric pollution”:ti,ab,kw
[3]	( PM_2.5_[Title/Abstract] OR Particulate Matter2.5[Title/Abstract] OR particulate matter[MeSH Terms] OR particulate matter[Title/Abstract] OR PM_10_[Title/Abstract] OR Particulate Matter10[Title/Abstract] OR SO_2_[Title/Abstract] OR Sulfur dioxide[MeSH Terms] OR Sulfur dioxide[Title/Abstract] OR NO_2_[Title/Abstract] OR Nitrogen dioxide[MeSH Terms] OR Nitrogen dioxide[Title/Abstract] OR NO_x_[Title/Abstract] OR Nitrogen oxides[MeSH Terms] OR Nitrogen oxides[Title/Abstract] OR O_3_[Title/Abstract] OR ozone[MeSH Terms] OR ozone[Title/Abstract] OR CO[Title/Abstract] OR Carbon monoxide[MeSH Terms] OR Carbon monoxide[Title/Abstract] OR Smog[MeSH Terms] OR Smog[Title/Abstract] OR black carbon[MeSH Terms] OR black carbon[Title/Abstract])	(TS=( “PM_2.5_” OR “Particulate Matter2.5” OR “particulate matter” OR “PM_10_” OR “Particulate Matter 10” OR “SO_2_” OR “Sulfur dioxide” OR “NO_2_” OR “Nitrogen dioxide” OR “NO_x_” OR “Nitrogen oxides” OR “O_3_” OR “ozone” OR “CO” OR “Carbon monoxide” OR “Smog” OR “black carbon”) OR TI=( “PM_2.5_” OR “Particulate Matter2.5” OR “particulate matter” OR “PM_10_” OR “Particulate Matter 10” OR “SO_2_” OR “Sulfur dioxide” OR “NO_2_” OR “Nitrogen dioxide” OR “NO_x_” OR “Nitrogen oxides” OR “O_3_” OR “ozone” OR “CO” OR “Carbon monoxide” OR “Smog” OR “black carbon”))	TITLE-ABS-KEY(“conjunctivitis” OR “endophthalmitis” OR “ophthalmia” OR “pinkeye” OR “conjunctivitis” OR “Pink eye”) AND TITLE-ABS-KEY(“air pollution” OR “ambient air pollution” OR “outdoor air pollution” OR “atmospheric pollution”) AND TITLE-ABS-KEY( “PM_2.5_” OR “Particulate Matter2.5” OR “particulate matter” OR “PM_10_” OR “Particulate Matter 10” OR “SO_2_” OR “Sulfur dioxide” OR “NO_2_” OR “Nitrogen dioxide” OR “NO_x_” OR “Nitrogen oxides” OR “O_3_” OR “ozone” OR “CO” OR “Carbon monoxide” OR “Smog” OR “black carbon”)	“PM_2.5_”:ti,ab,kw OR “particulate matter2.5”:ti,ab,kw OR “particulate matter”:ti,ab,kw OR “PM_10_”:ti,ab,kw OR “particulate matter 10”:ti,ab,kw OR “SO_2_”:ti,ab,kw OR “sulfur dioxide”:ti,ab,kw OR “NO_2_”:ti,ab,kw OR “nitrogen dioxide”:ti,ab,kw OR “NO_x_”:ti,ab,kw OR “nitrogen oxides”:ti,ab,kw OR “O_3_”:ti,ab,kw OR “ozone”:ti,ab,kw OR “CO”:ti,ab,kw OR “carbon monoxide”:ti,ab,kw OR “smog”:ti,ab,kw OR “black carbon”:ti,ab,kw
Search strategy	([1] AND [2]) OR ([1] AND [3])	([1] AND [2]) OR ([1] AND [3])	([1] AND [2]) OR ([1] AND [3])	([1] AND [2]) OR ([1] AND [3])

**Table A2:** Supplementary information of the included literature.

Study	Location	Population	GDP (billion dollars)	Latitude, Longitude	Temperature (°C)	Humidity (%)	Duration of Sunshine (hours)
Bourcier et al. (2003) [13]	Paris, France	2,125,851	459.20	48.86, 2.35	9.31–16.90	54.70–89.90	4.54
Larrieu et al. (2009) [14]	Bordeaux, France	600,000	17.70	44.84, −0.58	—	—	5.57
Chang et al. (2012) [4]	Taiwan, China	23,037,031	392.92	25.03, 121.52	24.09	75.24	5.26
Chiang et al. (2012) [29]	Taiwan, China (four cities) ^a^	22,689,122	331.01	25.03, 121.52	23.78	77.25	4.95
Szyszkowicz et al. (2012) [27]	Edmonton, Canada	626,500	28.80	53.53, -113.50	3.90	66.00	6.40
Hong et al. (2016) [6]	Shanghai, China	23,030,000	244.90	31.27, 121.52	17.20	69.40	4.88
Szyszkowicz et al. (2016) [28]	Ontario, Canada (nine cities) ^b^	12,760,000	657.20	50.00, -85.00	9.09	72.20	5.64
Fu et al. (2017) [5]	Hangzhou, China	9,018,000	145.93	30.25, 120.17	17.90	74.60	4.69
Jamaludin et al. (2017) [30]	Johor Bahru, Malaysian	848,000	20.06	1.46, 103.76	25.50–27.80	—	5.75
Lee et al. (2018) [11]	Daegu, Korea	2,279,000	45.387	35.87, 128.60	—	—	6.20
Seo et al. (2018) [10]	Seoul, South Korea	10,442,426	280.00	37.53,127.02	(7–9 month): 24.70 (1–3 month): −0.80	(7–9 month): 70.70 (1–3 month): 51.20	5.67
Szyszkowicz et al. (2019) [9]	Edmonton, Canada	626,500	28.8025	53.53, -113.50	—	—	6.40

Note: “—“, no data; “a”, Four cities included Taipei, Kaohsiung, Yunlin, and Yilan; “b”, Nine cities include Algoma, Halton, Hamilton, London, Ottawa, Peel, Toronto, Windsor, and York.

**Table A3:** The quality assessment of the included literature.

No.	Study	Conjunctivitis Disease Occurrence Verification (1 point)	Quality of Air Pollutant Measurement (1 point)	Adjustment Degree of Confounders (3 point)	Total Score (5 point)	Quality Category
1	Bourcier et al. (2003) [13]	0	1	3	4	Low quality
2	Larrieu et al. (2009) [14]	1	1	2	4	Medium quality
3	Chang et al. (2012) [4]	1	1	2	4	Medium quality
4	Chiang et al. (2012) [29]	1	1	3	5	High quality
5	Szyszkowicz et al. (2012) [27]	1	1	2	4	Medium quality
6	Hong et al. (2016) [6]	1	1	3	5	High quality
7	Szyszkowicz et al. (2016) [28]	1	1	2	4	Medium quality
8	Fu et al. (2017) [5]	1	1	1	3	Medium quality
9	Jamaludin et al. (2017) [30]	0	0	2	2	Low quality
10	Lee et al. (2018) [11]	1	0	0	1	Low quality
11	Seo et al. (2018) [10]	1	0	1	2	Low quality
12	Szyszkowicz et al. (2019) [9]	1	1	1	3	Medium quality

**Table A4:** Sensitivity meta-analysis using the leave-one-out method.

Literature	RR(95% CI)	Z-test	*p-*value	Q-test	Q-*p*	*τ^2^*	*I^2^*	*H^2^*
CO-3	1.0010(0.9990-1.0030)	2.747	0.006	0.000	1.000	0.000000	0.000	1.000
CO-8	1.0000(1.0000-1.0000)	0.656	0.512	0.000	1.000	0.000000	0.000	1.000
PM_10_-1	1.0030(0.9971-1.0089)	0.873	0.382	16.265	0.006	0.000031	70.778	3.422
PM_10_-2	1.0030(0.9971-1.0089)	1.052	0.293	14.681	0.012	0.000020	66.979	3.028
PM_10_-3	1.0040(0.9962-1.0119)	1.129	0.259	17.34	0.004	0.000040	62.007	2.632
PM_10_-4	1.0050(1.0011-1.0090)	2.293	0.022	10.376	0.065	0.000009	40.147	1.671
PM_10_-6	1.0030(0.9971-1.0089)	1.006	0.314	17.192	0.004	0.000033	74.577	3.933
PM_10_-8	1.0020(0.9961-1.0079)	0.757	0.449	14.793	0.011	0.000025	64.491	2.816
PM_10_-10	1.0030(0.9971-1.0089)	1.226	0.220	13.695	0.018	0.000024	70.384	3.377
SO_2_-1	1.0060(0.9923-1.0199)	0.789	0.430	47.145	0.000	0.000193	86.342	7.322
SO_2_-3	1.0010(0.9835-1.0188)	0.155	0.877	24.433	0.000	0.000287	78.076	4.561
SO_2_-4	1.0131(1.0091-1.0171)	5.303	0.000	11.336	0.045	0.000002	3.03	1.031
SO_2_-6	1.0030(0.9835-1.0229)	0.330	0.742	48.521	0.000	0.000345	90.725	10.782
SO_2_-7	1.0030(0.9835-1.0229)	0.268	0.789	48.338	0.000	0.000348	90.073	10.073
SO_2_-8	1.0020(0.9883-1.0158)	0.224	0.823	45.144	0.000	0.000160	83.838	6.187
SO_2_-10	1.0060(0.9923-1.0199)	0.887	0.375	42.562	0.000	0.000180	85.515	6.904
PM_2.5_-3	1.0050(0.9972-1.0129)	1.051	0.293	4.856	0.088	0.000033	60.187	2.512
PM_2.5_-6	1.0000(1.0000-1.0000)	3.459	0.001	6.228	0.044	0.000000	0.000	1.000
PM_2.5_-7	1.0040(0.9942-1.0139)	0.904	0.366	5.827	0.054	0.000042	65.186	2.872
PM_2.5_-8	1.0000(1.0000-1.0000)	-0.527	0.598	3.121	0.210	0.000000	36.356	1.571
NO_2_-1	1.0274(1.0094-1.0457)	2.943	0.003	23.445	0.000	0.000308	77.501	4.445
NO_2_-2	1.0315(1.0134-1.0498)	3.501	0.000	24.636	0.000	0.000293	76.202	4.202
NO_2_-3	1.0356(1.0195-1.0520)	4.463	0.000	8.329	0.139	0.000135	41.196	1.701
NO_2_-6	1.0222(1.0083-1.0364)	3.020	0.003	14.466	0.013	0.000158	61.183	2.576
NO_2_-7	1.0294(1.0094-1.0498)	2.779	0.005	24.110	0.000	0.000393	76.376	4.233
NO_2_-8	1.0263(1.0064-1.0467)	2.591	0.010	17.646	0.003	0.000341	72.691	3.662
NO_2_-9	1.0294(1.0114-1.0477)	3.392	0.001	24.980	0.000	0.000298	77.506	4.446
O_3_-1	1.0090(1.0031-1.0150)	2.783	0.005	48.211	0.000	0.000053	94.372	17.768
O_3_-2	1.0070(1.0011-1.0130)	2.802	0.005	44.353	0.000	0.000032	90.828	10.903
O_3_-3	1.0101(1.0022-1.0180)	2.622	0.009	40.214	0.000	0.000080	95.42	21.834
O_3_-4	1.0111(1.0032-1.0190)	2.793	0.005	39.621	0.000	0.000074	91.511	11.78
O_3_-5	1.0050(1.0011-1.0090)	2.982	0.003	38.258	0.000	0.000013	80.072	5.018
O_3_-6	1.0070(1.0011-1.0130)	2.648	0.008	42.643	0.000	0.000033	91.017	11.132
O_3_-7	1.0111(1.0032-1.0190)	2.745	0.006	43.741	0.000	0.000076	94.879	19.529
O_3_-8	1.0101(1.0022-1.0180)	2.689	0.007	47.910	0.000	0.000077	95.903	24.405
O_3_-11	1.0111(1.0051-1.0170)	3.218	0.001	16.862	0.018	0.000050	83.291	5.985

Note. The number in the column of “literature” denotes the number of the literature from Table A3 that was excluded, and effect estimates from the rest of the literature were then pooled using a meta-analysis. Q-p denotes the *p*-value for the Q test.
